# Diffusion-Limited Growth of Microbial Colonies

**DOI:** 10.1038/s41598-018-23649-z

**Published:** 2018-04-16

**Authors:** Hayden Tronnolone, Alexander Tam, Zoltán Szenczi, J. E. F. Green, Sanjeeva Balasuriya, Ee Lin Tek, Jennifer M. Gardner, Joanna F. Sundstrom, Vladimir Jiranek, Stephen G. Oliver, Benjamin J. Binder

**Affiliations:** 1 School of Mathematical Sciences, University of Adelaide, Adelaide, SA 5005 Australia; 2 Department of Wine and Food Science, Waite Campus, University of Adelaide, Urrbrae, SA 5064 Australia; 3 Cambridge Systems Biology Centre and Department of Biochemistry, University of Cambridge, Cambridge, CB2 1GA United Kingdom

## Abstract

The emergence of diffusion-limited growth (DLG) within a microbial colony on a solid substrate is studied using a combination of mathematical modelling and experiments. Using an agent-based model of the interaction between microbial cells and a diffusing nutrient, it is shown that growth directed towards a nutrient source may be used as an indicator that DLG is influencing the colony morphology. A continuous reaction–diffusion model for microbial growth is employed to identify the parameter regime in which DLG is expected to arise. Comparisons between the model and experimental data are used to argue that the bacterium Bacillus subtilis can undergo DLG, while the yeast Saccharomyces cerevisiae cannot, and thus the non-uniform growth exhibited by this yeast must be caused by the pseudohyphal growth mode rather than limited nutrient availability. Experiments testing directly for DLG features in yeast colonies are used to confirm this hypothesis.

## Introduction

**Figure 1:**
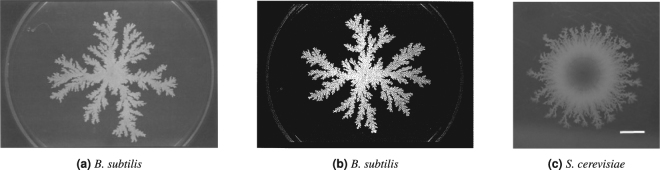
Experimental examples of microbial colonies. Shown are colonies of (**a**) *B*. *subtilis*^12^, (**b**) *B*. *subtilis*^1^, and (**c**) *S*. *cerevisiae*^31^. The petri dishes shown (a and b) were 88 mm in diameter, while the scale bar (**c**) represents 2 mm. The growth at the edge of the *S*. *cerevisiae* colony closely resembles that of the *B*. *subtilis* colonies. Figure 1(a) is reprinted from *Physica A: Statistical Mechanics and its Applications*, 168, Mitsugu Matsushita and Hiroshi Fujikawa, Diffusion-limited growth in bacterial colony formation, 498–506, 1990, with permission from Elsevier. Figure 1(b) is reproduced with permission from The Physical Society of Japan (*J. Phys. Soc. Jpn.* 58, 3875–3878, 1989.)

When placed on a solid substrate, many types of unicellular microbes, such as bacteria and fungi, grow into colonies consisting of numerous individual cells. The morphology of such a colony is highly dependent on the availability of nutrients that diffuse throughout the substrate. When sufficient nutrient is available, the cells grow into colonies that are uniform in shape^1,2^. When nutrients are limited, the colony may undergo diffusion-limited growth (DLG), which typically manifests as a non-uniform colony shape. While such a change can occur without any change in the morphology of individual cells, as exhibited by the bacterium *Bacillus subtilis*, certain microbes may also respond actively to low nutrient levels. Dimorphic yeasts, such as *Saccharomyces cerevisiae* (baker’s yeast), react to limited nutrient by switching to the pseudohyphal growth mode^2^, which consists of three features: a change in the cell budding pattern from the axial or bipolar modes to distal unipolar budding; an elongation of the cells; and the ongoing adhesion of mother and daughter cells. This response alters the colony morphology and allows the non-motile yeast cells to forage for nutrients^3^. Despite this difference, colonies of *B*. *subtilis* and *S*. *cerevisiae* grown in low-nutrient environments are strikingly similar in shape, as illustrated by the representative images shown in Fig. 1. It is not known whether yeast colony morphology is controlled by the pseudohyphal growth pattern or simply a consequence of DLG.

Microorganisms are ubiquitous in the environment, they play essential roles in the biogeochemical cycles^4^, are major agents of disease, and represent living factories for the production of fermented food and in biotechnology^5^. Accordingly, there is great interest in industrial strains with superior properties, such as improved fermentation rates, as well as preventing the persistence of unwanted strains via biofilm formation in process tanks or on medical devices. It is thus of interest to quantify how changes to microbe cell morphology manifest in the observed colony morphology, which requires an understanding of the transitions leading to different growth modes. Mathematical modelling provides a useful framework for identifying the key mechanisms involved and predicting the colony morphology. In particular, mathematical models afford a theoretical insight into the relationship between nutrient diffusion and colony morphology that augments experimental observations.

The pioneering studies of Pirt^6^ and Cooper *et al*.^7^ revealed that, between 12 and 24 hours after seeding, some microbial colonies cease growing with the expected exponential increase in mass and, instead, display a constant increase in radius. It was proposed that, during this period, the colony consumes nutrient faster than the nutrient is able to equilibrate, resulting in concentration gradients within the medium. Because of this, nutrient is only able to be consumed in a small region at the edge of the colony, which represents a form of DLG. Using this assumption, a one-dimensional continuum model of the radius was able to produce the expected constant growth. Following this phase, the area, rather than the radius, displayed a constant rate of increase, corresponding to a reduction in size of the growth region at the colony boundary.

Discrete lattice-based models have been used extensively to understand pattern formation in cell colonies, due largely to an ability to efficiently represent the observed complex morphologies^8,9^. Eden^10^ introduced a simple model for colony growth on a lattice in which new cells are added randomly to any vacant site adjacent to an existing cell. This represents growth occurring while nutrient is readily available and results in uniform morphologies, which is sometimes referred to as Eden-like growth. The aggregation of metal particles has been studied using the related diffusion-limited aggregation (DLA) model introduced by Witten & Sander^11^. In this model, each new cell is placed at a random location and then performs a random walk on the lattice until reaching a site adjacent to an existing cell, at which point the cell is fixed. The cell performing the random walk may instead be interpreted as a nutrient particle that is absorbed by an existing cell upon contact, allowing the existing cell to reproduce and place a new cell in an adjacent site. Under this interpretation, the DLA model represents colony growth at very low nutrient concentrations and thus represents a form of DLG. Simulations using this model suggested that outer branches ‘screen’ inner sites and consequently grow faster.

Colonies of *B*. *subtilis* grown on an agar substrate have also been observed to develop short branches next to longer branches, which indicates that the longer branches are ‘screening’ the shorter branches from some diffusing quantity^1^. Additional experiments by Matsushita & Fujikawa^12,13^ replicated this behaviour, while it was further observed that two colonies seeded close together in a uniform nutrient field grew in opposite directions, as if repelling, and a single colony placed in the centre of a petri dish grows towards a nutrient source placed on one side only. The occurrence of these three characteristic features was used to conclude that DLG was controlling the morphology. A number of studies have modelled bacterial colony growth using either coupled reaction-diffusion systems^14–20^, agent-based models^21–24^ or a combination of discrete and continuous approaches^25,26^, producing colony morphologies that closely resemble experimental bacterial colonies exhibiting branch screening, while similar models have also been applied to biofilms^27–29^. In particular, Ginovart *et*. *al*.^30^ used a lattice-based model of bacterial growth to reproduce the three features of bacterial-colony growth observed by Matsushita & Fujikawa^12^, demonstrating that this behaviour arises due to DLG alone. While this work went some way towards quantifying colony growth, no attempt was made to quantify the behaviour of repelling colonies or directed growth. A limited number of studies have modelled the growth of yeast colonies specifically^31–34^; however, the extent to which DLG influences yeast colony morphology has not yet been thoroughly investigated.

While the cell aspect ratio of dimorphic yeast provides a visual indication that the colony has entered the pseudohyphal growth mode, the low resolution of typical experimental images means that the cell aspect ratios are generally not known and thus it is not even possible to identify whether a yeast colony has entered the pseudohyphal growth mode from a single image alone. Previous mathematical studies have shown that the morphology of non-uniform yeast colonies can be reproduced using either DLG or the pseudohyphal budding pattern^31^. There is thus a need to develop a framework for identifying the dominant growth mechanisms that does not rely on observations of individual cells. While experimental observations of non-motile *B*. *subtilis* have shown that DLG only occurs at sufficiently low nutrient concentrations and that higher nutrient concentrations result in Eden-like (uniform) growth^35^, these classifications have been based on qualitative observations only, and no quantitative classification or modelling has been undertaken to determine the nutrient concentration at which this transition occurs.

We seek further understanding of DLG in microbial colonies by using mathematical models to identify the conditions under which DLG is expected to occur. The lattice-based model of microbial growth introduced by Matsuura^23^ is adopted as it explicitly simulates the interaction between cells and diffusing nutrient, and is suited to the complex patterns that arise during DLG. The model is used to replicate the experiments of Matsushita & Fujikawa^12^ so as to quantify the growth patterns of the three observed DLG phenomena and determine how these are influenced by nutrient availability. This analysis shows that directed growth is a useful indicator of DLG. We then use this result, in conjunction with a continuous model comprising a coupled system of reaction–diffusion equations, to argue that the emergence of DLG depends upon two dimensionless parameters: the ratio of the microbe and nutrient diffusivities; and the dimensionless initial nutrient concentration, the latter of which depends on the cell proliferation rate. Being deterministic, this model allows both of these quantities to be specified precisely. Using the established regimes, we examine representative experimental examples of microbial colonies to identify which colony morphologies are likely to have arisen due to DLG and, ultimately, which species are influenced by this effect.

## Results

### Discrete model

The growth of a microbial colony that is consuming a diffusing nutrient is represented using the lattice-based model introduced by Matsuura^23^, the details of which are given in section 3. Briefly, we consider a rectangular lattice with *L*_*x*_ and *L*_*y*_ sites in the *x*- and *y*-directions, respectively. The number of occupied cells is denoted *ν* with corresponding cell density *ρ* = *ν*/(*L*_*x*_*L*_*y*_). Each element of the rectangular lattice may house at most one cell but may hold any non-negative integer number of nutrient particles, regardless of whether there is also a cell at that site. At each time step, the yeast cells may absorb a nutrient particle located at the same site and produce a single daughter cell in an adjacent site in a cardinal direction, while nutrient particles may take *s* steps, again in the cardinal directions. As an initial condition, cells are seeded within the lattice in a prescribed pattern, while a number of nutrient particles are placed uniformly at random within the domain to give a specified average initial concentration *c*_0_. The boundaries of the domain are treated as solid walls, replicating the experimental behaviour within a petri dish. Importantly, the patterns produced by this model are the result of the interaction between the cells and nutrient alone, so that any non-uniform morphologies produced by the model may be attributed entirely to DLG.

### Characteristic DLG morphologies

**Figure 2:**
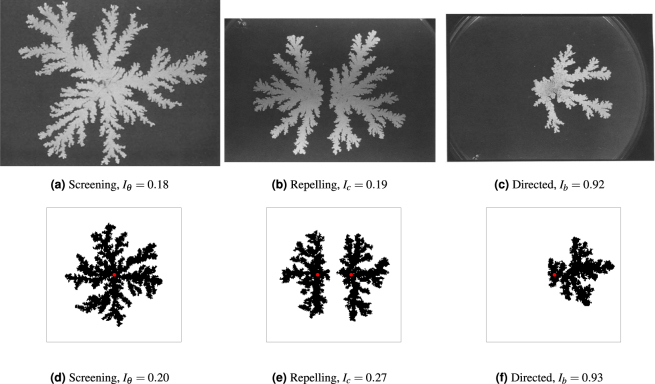
Experimental results by Matsushita & Fujikawa^12^ (top row) with corresponding model simulations (bottom row). Shown are (**a**) larger branches screening smaller branches from nutrient (phenomenon I), (**b**) two colonies seeded close together appearing to repel each other (phenomenon II), and (**c**) a single colony growing towards the nutrient source on the right-hand side of the petri dish (phenomenon III). Simulations of (**d**) screening branches, (**e**) two colonies in close proximity and (**f**) growth with nutrient on the right-hand side are computed using the lattice-based model. The seed cells are marked by a red dot. The simulations were computed on lattices with dimensions *L*_*x*_ = *L*_*y*_ = 200 with parameters *s* = 3 and *c*_0_ = 1, which result in a value of Δ of the same order of magnitude as in the experiments. Figures 2(a), 2(b) and 2(c) are reprinted from *Physica A: Statistical Mechanics and its Applications*, 168, Mitsugu Matsushita and Hiroshi Fujikawa, Diffusion-limited growth in bacterial colony formation, 498–506, 1990, with permission from Elsevier.

Matsushita & Fujikawa^12^ used a colony of *B*. *subtilis* cells to illustrate three key phenomena that arise due to DLG: (I) the ‘screening’ of shorter branches by longer ones; (II) repulsion between neighbouring colonies; and (III) growth directed towards a nutrient source (Fig. 2). These features have previously been shown to arise due to DLG alone using a lattice-based model of the colony growth similar to that used here^30^. We first confirm that the discrete model described above can reproduce this behaviour before using this model to quantify the patterns produced.

The interaction between the cells and nutrient may be quantified broadly by comparing the relative rate of spread of these two quantities. The colony growth is measured by computing the average rate of change in the colony area Δ_*m*_ when viewed from above, which has the same units as a diffusivity. This quantity is calculated readily from an experimental image or from simulated data, such as that produced by the discrete model. The spread of the nutrient Δ_*n*_ is taken to be the diffusivity of glucose as this is commonly used as a nutrient source and there is little difference between the diffusivity of different nutrients. The diffusivity of glucose in water is known to be approximately ${D}_{0}=4.03\times {10}^{-2}$mm^2^ min^−1^, based upon experimental observations^36^. For glucose in a low-density agar gel, the diffusivity is given by

1 $$D=\mathrm{(1}-2.3w){D}_{0},$$

where *w* is the percentage by weight of agar^37^. Assuming *w* = 0.3%, the diffusivity is 4.01×10^−2^ mm^2^ min^−2^, which is the value used for the remainder of this study. The diffusivity changes little with the amount of agar, and hence *w* has a negligible impact on the results. From these quantities we compute the ratio

2 $${\rm{\Delta }}=\frac{{{\rm{\Delta }}}_{m}}{{{\rm{\Delta }}}_{n}},$$

which provides a dimensionless measure of the relative rates of spread. At small values of Δ, nutrient diffuses on a faster time scale than the cell growth, which means that any local variations in the nutrient concentration will dissipate without influencing the colony morphology. A value of Δ that is 1 or larger indicates that the cell growth is occurring at a rate at least as large as the nutrient diffuses, and local variations in the nutrient concentration may have an impact on the colony morphology. Of the three experimental images, only the image of directed growth has sufficient information provided on both the scale and time required to compute Δ. From this image we find that Δ ≈ 0.23. Setting *s* = 3 and *c*_0_ = 1 in the model produces solutions with values of Δ between 0.3 and 0.5, which is of the same order of magnitude as the experimental results and thus provides a suitable comparison. These parameter values are used for the remainder of this subsection. The behaviour in each of the three cases is further quantified using spatial indices, described below, similar to the approach of Binder *et al*.^38^. Each simulation uses a lattice with dimensions *L*_*x*_ = *L*_*y*_ = 200, which is large enough to produce features with sufficient resolution while still being computationally efficient.

To examine branch screening (phenomenon I), nutrient is seeded uniformly at random across the domain and a single cell is placed in the centre of the lattice. The simulation is run until the total cell density reaches 0.2, illustrated by the representative colony shown in Fig. 2. This colony features large branches emanating from the site of the initial central cell, with shorter branches in between that have been screened from nutrient by the larger branches, and displays significant non-uniform growth. This matches the behaviour observed by Matsushita & Fujikawa^12^. The morphology may be quantified by first counting the angles to each cell measured counter-clockwise from some reference angle with the origin placed at the centre of mass. The counts are scaled by the expected values for cells distributed uniformly at random, and the angular index of non-uniform growth *I*_*θ*_ is defined to be the standard deviation of the scaled counts, so that larger values of *I*_*θ*_ indicate greater levels of non-uniform growth. The experimental image has index 0.18, while the simulation has index 0.2, indicating that the two are in close agreement.

For the case of repelling colonies (phenomenon II), nutrient is again placed uniformly at random across the domain. Two seed cells are placed vertically centred within the domain, each an eighth of the domain width away from the centre horizontally so that the cells are separated by one quarter of the total domain width. The simulation is computed until the total cell density reaches 0.2. A typical simulation is shown in Fig. 2, which displays the gap between the two colonies observed by Matsushita & Fujikawa^12^. Repelling colonies may be quantified by counting the total number of cells *ν* and the number of cells *ν*_*c*_ between the two seed cells at the end of the simulation. The index of repulsion is then defined to be *I*_*c*_ = 1 − *ν*_*c*_/*ν*, which is close to 0.5 when the two colonies grow uniformly, smaller than 0.5 when a gap forms, and greater than 0.5 when the colonies show a preference for growth towards each other. The seed cell locations for each colony in the experimental image are approximated by drawing lines along the branches and identifying where these intersect. The experimental image and simulation have indices 0.19 and 0.27, respectively, which suggests that both are producing similar growth patterns with a significant gap between the two colonies.

Directed growth (phenomenon III) is simulated by initially placing all nutrient in the rightmost column of the domain, with a single cell placed in the centre of the domain. The simulation is then computed until the cell density reaches 0.1. A typical colony is shown in Fig. 2, which closely resembles the experimental result of Matsushita & Fujikawa^12^. To measure bias towards one side of the domain we compute the proportion *I*_*b*_ of cells on the right-hand side of the domain relative to the total number of cells, so that *I*_*b*_ ∈ [0,1]. Values of the index *I*_*b*_ close to 0.5 indicate little bias, while *I*_*b*_ < 0.5 indicates bias towards the right-hand side and *I*_*b*_ < 0.5 indicates bias towards the left. The experimental image has index 0.92, which closely matches the simulation index of 0.93. In both cases the indices indicate a large growth bias towards the initial nutrient location.

As found by Ginovart *et al*.^30^, the good qualitative matches between the experimental images and the simulations show that DLG alone can produce the screening, repulsion, and directed growth of *B*. *subtilis* colonies. We have further strengthened this comparison through the use of a quantitative comparison between the experiments and a mathematical model. Thus, we expect these phenomena to be present when DLG is influencing the morphology, while the absence of these features suggests that other mechanisms are responsible for the growth pattern. Crucially, the agreement between the discrete model and the model proposed by Ginovart *et al*. demonstrates that the discrete model employed here provides a satisfactory representation of DLG and thus may be used to quantify this behaviour.

### Inducing DLG

Having shown that the discrete model is able to replicate DLG behaviour, we here quantify the dependence of these phenomena on the model parameters so as to predict when DLG effects will arise. Colonies are again simulated on a lattice with dimensions *L*_*x*_ = *L*_*y*_ = 200 using the same three initial conditions and stopping criteria as in the previous subsection. Simulations are repeated 50 times for each pair of nutrient steps *s* = 1, 5, …, 37 and initial concentrations *c*_0_ = 1, 2, …, 7. For each parameter pair we compute the relevant average index over the 50 realisations.

**Figure 3:**
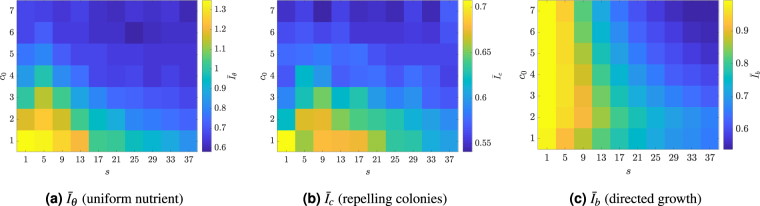
Measures of DLG effects in simulated microbial colonies. All simulations are computed using the lattice-based model using a range of nutrient steps *s* and initial nutrient concentrations *c*_0_. Shown are (**a**) the mean index ${\bar{I}}_{\theta }$ for branch screening (phenomenon I), (**b**) the mean index ${\bar{I}}_{c}$ for repelling colonies (phenomenon II), and (**c**) the mean index ${\bar{I}}_{b}$ for directed growth (phenomenon III).

To examine branch screening (phenomenon I), we consider colonies grown from a single cell in a uniform nutrient field, with the corresponding values mean index values ${\bar{I}}_{\theta }$ over the 50 realisations shown in Fig. 3. The largest values of ${\bar{I}}_{\theta }$ arise when both *s* and *c*_0_ are small, which is due to two factors. Firstly, because the nutrient diffusivity, effectively *s*, is small relative to the rate of cell growth, fluctuations in the nutrient levels develop across the domain. Secondly, the low initial nutrient concentration *c*_0_ means that these fluctuations create regions in which the nutrient level is too low to support cell growth. When either *s* or *c*_0_ is larger, at least one of these conditions cannot occur and the value of ${\bar{I}}_{\theta }$ becomes smaller, indicating that DLG no longer has a significant influence on the colony. Thus, these results indicate that non-uniform patterns can only occur when both the nutrient diffusivity, relative to the rate of cell growth, and the nutrient concentration are small.

Similar behaviour is observed for the repelling case (phenomenon II), as seen from the average index ${\bar{I}}_{c}$ plotted in Fig. 3. The largest values of the index are found at small values of *s* and *c*_0_, which occurs for the same reasons as for ${\bar{I}}_{\theta }$.

The behaviour for directed growth (phenomenon III) is different, as seen from the average index ${\bar{I}}_{b}$ shown in Fig. 3. At small values of *s*, the index ${\bar{I}}_{b}$ is large and varies little with *c*_0_. As *s* increases, ${\bar{I}}_{b}$ decreases and shows greater dependence on *c*_0_, with larger values of ${\bar{I}}_{b}$ at lower *c*_0_. The range of parameter values over which directed growth may be observed is much larger than for the other two DLG phenomena. Therefore, if directed growth does not occur then neither will the other two features, and thus directed growth provides a useful first check for DLG that is simple to measure. This feature will be used to test for DLG throughout the remainder of this work.

### Continuum model

The emergence of DLG phenomena depends on both the nutrient concentration and the diffusivities of the two species. While the indices measure the dependence of these phenomena on the number of discrete nutrient steps *s* and initial nutrient concentration *c*_0_, the value of Δ could only be computed from the simulated data rather than specified as input to the model. When considering experimental data, however, it is natural to characterise the relative spread of the cells and nutrient using Δ, as this may be measured readily from experimental images. We here introduce a deterministic system of reaction–diffusion equations that model the cell density and nutrient concentration and allows the specification of the relative diffusivities of each quantity, which is equivalent to setting Δ. While this model is not suited to capturing the fine features observed in Fig. 2, it is able to replicate directed growth towards a nutrient source (phenomenon III), which was found to arise over the largest range of parameters. We therefore focus on this aspect of DLG, which acts as an easily measurable sign that DLG is occurring.

We consider a one-dimensional domain, which is sufficient to illustrate the general behaviour of the model^29,32,39^. Using the dimensionless position *x*, time *t*, cell density *n*(*x*,*t*) and nutrient concentration *g*(*x*, *t*), described in section 3, the governing equations reduce to

3a $$\frac{\partial m}{\partial t}=D\frac{{\partial }^{2}m}{\partial {x}^{2}}\,+mn,$$

3b $$\frac{\partial n}{\partial t}=\frac{{\partial }^{2}n}{\partial {x}^{2}}-\,cmn\mathrm{.}$$

The parameter *D* = *D*_*m*_/*D*_*n*_ is the ratio of the cell diffusivity *D*_*m*_ to that of the nutrient *D*_*n*_. This is similar to the definition of Δ (2), using the cell diffusivity in place of the measured rate of change in the colony area. The first term on the right-hand side of both equations represents the contribution of diffusion, while the second terms represent the consumption of nutrient and growth of new cells, respectively, with *c* the dimensionless quantity of nutrient consumed per new cell.

**Figure 4:**
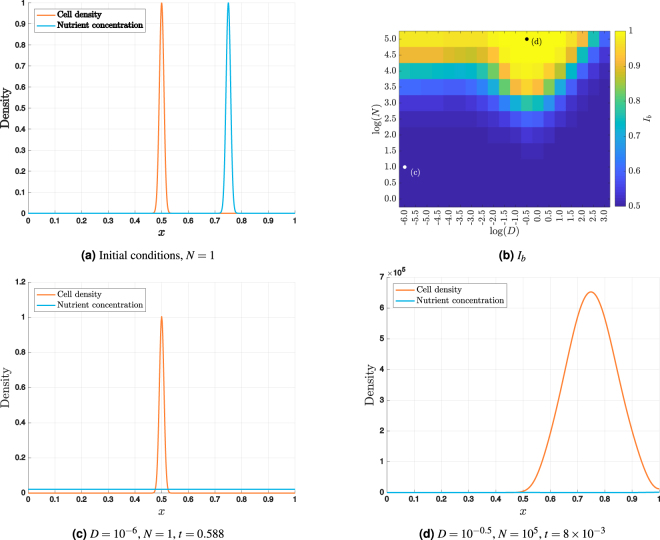
Results from the one-dimensional reaction–diffusion model. (**a**) The initial condition for *N* = 1 shows the cells concentrated in the centre of the domain with the nutrient to the right. (**b**) The maximum values of *I*_*b*_ up to time *t* = 1, plotted against the base 10 logarithm of *D* and *N*, suggest that DLG only occurs at particular parameter values. Two representative examples are shown marked. (**c**) At the the largest value of *I*_*b*_ when *D* = 10^−6^ and *N* = 1, the cells are still almost symmetrical about *x* = 0, while the nutrient concentration has become effectively uniform. (**d**) Using *D* = 10^−0.5^ and *N* = 10^5^ results in a significant bias towards the right-hand side, where the nutrient was initially concentrated.

As initial conditions, cells are placed in the centre of the domain with the nutrient biased to the right according to

4a $$m(x\mathrm{,0)}={e}^{-L{(x-0.5)}^{2}},$$

4b $$n(x\mathrm{,0)}=N{e}^{-L{(x-0.75)}^{2}},$$

where *N* may be interpreted as a dimensionless nutrient concentration. To illustrate the general behaviour, the initial conditions for *N* = 1 are shown in Fig. 4.

Taking the typical parameter values given in section 3 as fixed, the value of *N* only varies due to the physical nutrient concentration. Considering a medium containing only nutrient, representing a maximum concentration, we find that the value of *N* can be no greater than approximately 10^5^. Solutions are thus computed for 1 ≤ *N* ≤ 10^5^. While typical experimental observations suggest that 10^−3^ ≤ *D* ≤10^−1^, we consider values for 10^−6^ ≤ *D* ≤10^3^ so as to provide a theoretical examination of how the behaviour changes with *D*. For different values of these parameters, we compute the maximum value of *I*_*b*_ observed up until time *t* = 1. This corresponds to approximately 119 days of growth, which, while larger than typical experimental times, ensures that the maximum value of *I*_*b*_ is observed during the simulation. The computed indices *I*_*b*_ are plotted in Fig. 4 using a logarithmic scale in base 10 for both axes. For log(*N*) < 1, there is little or no bias in the growth of the cells, as measured by *I*_*b*_. At larger values of *N*, the amount of bias observed depends upon the value of *D*, with the maximum occurring near (*D*,*N*) = (1, 10^5^). This value of *D* corresponds to cell and nutrient diffusivities of equal magnitude and around this value it is possible to observe bias in the growth for values of *N* as small as 10^1.5^. Under typical experimental conditions, *N* has order of magnitude 2, which indicates that DLG effects are most likely to be observed when *D* is close to unity.

The range of behaviour is further illustrated by considering the distributions from two contrasting examples. In each case, the solution is shown at the time corresponding to the maximum cell bias. For *D* = 10^−6^ and *N* = 1, shown in Fig. 4, the nutrient concentration has become effectively uniform before the cell density can develop any obvious bias towards the right-hand side, where the nutrient was initially concentrated. In contrast, taking *D* = 10^−0.5^ and *N* = 10^5^, also plotted in Fig. 4, the cells show an obvious preference towards the right-hand side of the domain.

The analysis of the continuum model suggests that, if $D\ll 1$, then directed growth, and hence any DLG effects, will only occur at values of *N* at least as large as 10^3^. Since estimates indicate that *N* has order of magnitude 2, this suggests that DLG will only be observed when *D* is close to unity, as evident from Fig. 4. This may also be illustrated using physical parameters. Using the parameter values given in section 3, microbes with diffusivity ${D}_{m}=3\times {10}^{-2}$mm^2^ min^−1^ placed in an environment with maximum initial nutrient concentration ${N}_{0}\mathrm{=3.8}\times {10}^{-3}$g mm^−2^ would correspond approximately to the dimensionless values *D* = 0.75 and *N* = 10^4^. From Fig. 4, it would be expected that this species would grow towards a nutrient source and hence exhibit DLG behaviour. If the same microbes were placed in an environment with maximum nutrient concentration ${N}_{0}\mathrm{=3.8}\times {10}^{-6}$g mm^−2^, the value of *N* would drop to 10 and biased growth would no longer be observed. The results of this section thus provide a framework for identifying when DLG is expected to occur based only on estimates of *D* and *N*.

### Experimental comparisons

Having examined the model predictions, we now use these to identify the dominant growth mechanism in microbial colonies. We consider the three representative experimental examples shown in Fig. 1: two colonies of the bacterium *B*. *subtilis* and one colony of *S*. *cerevisiae*. As we do not know the appropriate value of the diffusion ratio *D*, which is required by the reaction–diffusion model, the growth is instead characterised by the relative spread Δ (2). This parameter represents the ratio of the average rate of change in the colony area, when viewed from above, to the diffusivity of glucose and can be measured from the images. As the nutrient is uniformly distributed and only a single colony is grown, any DLG in these examples is expected to manifest as irregular growth with branch screening (phenomenon I).

The computed values of the growth rates Δ_*m*_ are given in Table 1, along with the corresponding relative rates Δ. These values indicate that the *B*. *subtilis* colonies grow two orders of magnitude faster than the yeast colony and one order of magnitude slower than the diffusivity of glucose. Using typical values for the initial nutrient concentration suggests that the experiments correspond to a value of *N* that has order of magnitude 2. Matching this estimate and the values of Δ to the model results from Fig. 4 indicates that *B*. *subtilis* corresponds to a regime in which directed growth due to DLG will occur. As this estimate was made by measuring the cell proliferation rate *p* within a colony of *S*. *cerevisiae*, which is likely to be smaller than the corresponding value for bacteria, this is expected to be an underestimate for *N* and a larger value of *N* increases the likelihood of observing DLG. In contrast, the *S*. *cerevisiae* colony has a Δ with order of magnitude –3, which indicates that the nutrient diffuses on a much faster time scale than that of cell growth. As a consequence, any local variations in the nutrient concentration are expected to dissipate before having an influence on the colony morphology. This indicates that the morphology is not influenced by DLG. Thus, despite the strong resemblance between the shapes of bacterial and yeast colonies in low-nutrient environments, these two morphologies are due to different phenomena. Bacterial colonies grow on a sufficiently fast time scale that nutrient diffusion may limit the growth, resulting in an irregular pattern. The much slower growth of yeast colonies means that DLG cannot occur and, instead, the non-uniform colony morphologies arising in low-nutrient environments must be due to pseudohyphal growth alone.

**Table 1:** Estimated growth rates from the experimental data.

Colony	Day	Δ_*m*_(m^2^·s^−1^)	Δ	Source
*B*. *subtilis*	20	1.4×10^−10^	2.0×10^−1^	^1^p.3876
*B*. *subtilis*	21	1.0×10^−10^	1.6×10^−1^	^12^ p.502
*S*. *cerevisiae*	58	2.0×10^−12^	3.0×10^−3^	^31^p.10

**Figure 5:**
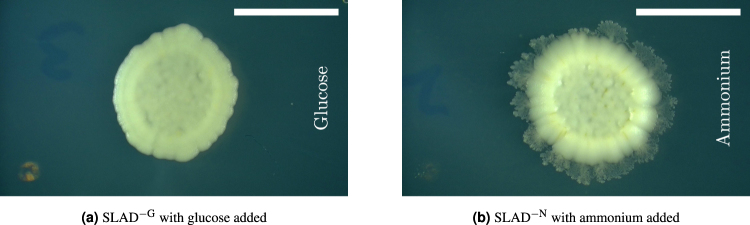
Images from the directed growth experiments using *S*. *cerevisiae*. The images are orientated so that the corresponding nutrient is on the right-hand side of the colony, as indicated by the vertical text. The colonies were grown on (**a**) SLAD^−G^ with glucose added to the right and (**b**) SLAD^−N^ with ammonium sulphate added to the right. The scale bars represent 5 mm.

We sought further confirmation of these results by testing for directed growth (phenomenon III) in colonies of *S*. *cerevisiae*, mimicking the set-up used by Matsushita & Fujikawa^12^ and in the simulations^40^. A petri dish was filled with synthetic low-ammonium dextrose (SLAD) with nutrient placed in the centre of the petri dish. Yeast cells were seeded at different distances from the centre and photographed after 16 days of growth. Further experimental details are given in section 3. Both glucose and ammonium sulphate were used as the limited nutrient, with representative images for each shown in Fig. 5. The images are orientated so that the centre of the petri dish, where the nutrient was placed, is on the right-hand side. The diffusivity of ammonium in water is approximately 9.84×10^−2^ mm^2^ min^−1^ ^41^. As this is of the same order of magnitude as the diffusivity of glucose, each nutrient source is expected to result in a similar value of Δ. Neither colony shows a noticeable bias in growth in any direction, and both produce bias indices *I*_*b*_ almost exactly equal to 0.5. The effective diffusivities Δ_*m*_ and dimensionless diffusivities Δ for each trial are given in Table 2. In both cases Δ has order of magnitude –3, which indicates that directed growth should not be observed and agrees with the results from the previous experiments.

**Table 2:** Estimated absolute and relative growth rates from the directed-growth experiment.

Colony	Day	Δ_*m*_(m^2^·s^−1^)	Δ
*S*. *cerevisiae*	16	3.8×10^−12^	5.8×10^−3^
*S*. *cerevisiae*	16	6.0×10^−12^	9.0×10^−3^

The colonies are shown in Fig. 5. In both examples, the value of Δ has order of magnitude –3, which suggests that directed growth will not occur. This agrees with the behaviour observed in the images.

Further evidence of the growth mode is provided by the behaviour near the edge of the colonies. There are clear signs of non-uniform growth around the boundary of the colony grown on SLAD^−N^ but not on the colony grown on SLAD^−G^. If this pattern was due to DLG, we would expect similar behaviour on both media. It is known, however, that diploid yeasts, like the AWRI796 strain used in this experiment, switch to pseudohyphal growth when deprived of nitrogen^2^, such as SLAD^−N^. This suggests that the non-uniform growth observed in yeast colonies, such as shown in Fig. 1, is due to pseudohyphal growth and not DLG.

## Discussion

The emergence of diffusion-limited growth in microbial colonies has been studied using a combination of mathematical modelling and experiments. An agent-based model for the interaction between cells and nutrient was used to replicate the three phenomena observed by Matsushita & Fujikawa^12^, namely branches with screening, repelling colonies, and directed growth. In each case, the simulations showed a close match to the experimental data when compared using spatial indices, and replicated the simulations of Ginovart *et al*.^30^. The model could thus be used to quantify the behaviour of each phenomenon, showing that biased growth provides a good indicator of DLG.

The cells and nutrient were then modelled as continuous fields using a system of reaction–diffusion equations. This model allowed both the ratio of the diffusivities and the dimensionless nutrient concentration to be specified precisely, and showed that directed growth towards a nutrient source could only occur at certain values of these parameters, which correspond to values of the physical parameters. By matching the model results to experimental data, it was found that the typical growth of the bacterium *B*. *subtilis* corresponds to a region in which directed growth due to DLG may occur, while that of the yeast *S*. *cerevisiae* does not. Experiments confirmed that yeast colonies grown in nutrient-limited environments do not produce growth towards a nutrient source like that exhibited by bacterial colonies. Furthermore, filamentous-like growth was only observed in yeast colonies grown in an initially nitrogen-free environment and not in colonies grown in nitrogen-limited and initially glucose-free environments. As it is known that dimorphic yeasts switch to the pseudohyphal growth mode when deprived of nitrogen^2^, this indicates that the irregular growth occurred due to the pseudohyphal growth mode and not DLG. These observations together support the conclusion that the non-uniform growth of yeast colonies is due to the pseudohyphal growth pattern adopted by the cells and is not influenced by DLG.

Binder *et al*.^38^ (Fig. 4c), observed that yeast colonies display a linear increase in radius up until the onset of filamentous growth, which resembles the linear growth phase displayed by other microbial colonies^7^. It has been proposed that the linear phase occurs due to gradients in the nutrient concentration that limit growth to a small region around the colony boundary^6^. The results of the present work suggest that, for yeast colonies, any such gradients should equilibrate on a much faster time scale than the cell growth rate and thus should not inhibit the growth. This indicates that the observed linear increase in yeast colony radius is the result of some other limiting factor, such as cell crowding in the centre of the colony or toxic byproducts produced by the cells. The experiments of Binder *et al*.^38^ also indicated that the colony radius increased at a slower rate during filamentous growth, resembling the area growth law in which the growth region at the colony boundary decreases in proportion to the radius. This has been attributed to the production of toxins during the growth, which is consistent with the above hypothesis^7^.

It has been suggested that irregular growth in yeast colonies is triggered simultaneously by signalling compounds^42,43^. Such compounds would diffuse at a similar rate to nutrient. Using the results of this analysis, on the time scale of the cell growth, yeast cells would receive these signals at effectively the same time. This agrees with experimental observations of pseudohyphal growth, such as shown in Fig. 1, which show this pattern occurring around the entire colony simultaneously.

While the nutrient diffusivity is typically known and the amount of nutrient consumed per new cell can be estimated with reasonable accuracy, it is difficult to compute the value of the cell proliferation rate, which is used to compute *N*. Since the other terms in this expression are usually well known, and provided *D* is known, it would be possible to conduct an experiment to measure the bias *I*_*b*_ and match this to the model results in order to determine the value of *N*, from which the value of the cell proliferation rate could be determined.

This study illustrates how multiple modelling methodologies, each with differing strengths and weaknesses, can work in concert to illuminate a biological process. The agent-based model is able to capture the fine details and complex patterns observed in DLG; however, this model is stochastic in nature and does not allow the specification of the diffusivity ratio. The reaction–diffusion model is deterministic and requires the specification of the diffusivity ratio, but cannot easily represent small features of a colony undergoing DLG without using a fine computational grid. By making use of both approaches, these limitations may be overcome and a complete picture of the physical behaviour may be found, augmenting experimental observations.

## Methods

### Discrete model rules

If a nutrient packet occupies the same element as a cell, then the cell absorbs the nutrient with some probability, and each cell may store at most 4 units of nutrient at any time. A cell may produce a daughter in any unoccupied site in one of the cardinal directions with probability *p*_*r*_ = 0.5. At each time step, a nutrient packet moves *s* units within the lattice with probability *p*_*m*_ = 0.8, again only in the cardinal directions. The value of *s* represents the number of steps each nutrient packet can move in the time it takes for a cell to reproduce.

### Reaction–diffusion model derivation

We consider a one-dimensional domain of width *L* with Cartesian co-ordinate *x* ∈ [0, *L*] and time *t*. The cell density is denoted *m*(*x*, *t*) and the nutrient concentration *n*(*x*, *t*). The two species evolve according to the governing reaction–diffusion equations

5a $$\frac{\partial m}{\partial t}={D}_{m}\frac{{\partial }^{2}m}{\partial {x}^{2}}+pmn,$$

5b $$\frac{\partial n}{\partial t}={D}_{n}\frac{{\partial }^{2}n}{\partial {x}^{2}}-cpmn,$$

where *D*_*m*_ and *D*_*n*_ are the cell and nutrient diffusivities, respectively, *p* is the cell proliferation rate, and *c* is the quantity of nutrient consumed per new cell^29,32,39^. The initial conditions are

6a $$m(x,\,\mathrm{0)}={M}_{0}{e}^{-{(x-0.5L)}^{2}},$$

6b $$n(x\mathrm{,0)}={N}_{0}{e}^{-{(x-0.75L)}^{2}},$$

where *M*_0_ and *N*_0_ are representative values of the initial cell density and nutrient concentration. We introduce dimensionless variables, denoted by hats, and dimensionless parameters defined by

7 $$\hat{m}=\frac{1}{{M}_{0}}m,\,\hat{n}=\frac{p{L}^{2}}{{D}_{n}}n,\,\hat{t}=\frac{{D}_{n}}{{L}^{2}}t,\,\hat{x}=\frac{1}{L}x,\,D=\frac{{D}_{m}}{{D}_{n}},\,N=\frac{p{L}^{2}{N}_{0}}{{D}_{n}},\,\hat{c}=\frac{cp{M}_{0}{L}^{2}}{{D}_{n}}.$$

Transforming to these dimensionless variables and dropping hats yields the dimensionless equations analysed in this study. Typical parameter values are given in Table 3. The value *L* = 83 mm is chosen as this represents the available width in a standard petri dish in which a colony can grow. The values of *p*, *c* and *M*_0_ are estimated from observations of an *S*. *cerevisiae* biofilm^29^, and yield $\hat{c}=1.32\times {10}^{-2}$. Since we are interested in varying the dimensionless diffusivity *D* and dimensionless concentration *N* we do not need to estimate values for these parameters. As such, we do not need to know values for the microbe diffusion coefficient *D*_*m*_ and representative nutrient concentration *N*_0_.

**Table 3:** Parameter values for the reaction–diffusion model.

Parameter	Symbol	Value	Unit
Cell proliferation rate	*p*	15.28	mm^2^ g^−1^ min^−1^
Nutrient per new cell	*c*	3.473×10^−11^	g cell^−1^
Nutrient diffusivity	*D*_*n*_	4.01×10^−2^	mm^2^ min^−1^
Domain length	*L*	83	mm
Maximum initial cell density	*M*_0_	144.509	cell mm^−2^

### Directed growth experiments

Both glucose and ammonium were considered as the restricted nutrient sources, requiring two SLAD formulations:

SLAD^−G^: SLAD without glucose, with final concentrations of 1.7g 1^−1^ yeast nitrogen base without amino acids or ammonium sulphate (Becton Dickinson) and 50 μM ammonium sulphate.

SLAD^−N^: SLAD without ammonium, with final concentrations of 1.7g 1^−1^ yeast nitrogen base without amino acids or ammonium sulphate and 2% glucose.

A 2× solution of each SLAD formulation was prepared in ultrapure water and filter sterilised. Bacto agar (4%) (Becton Dickinson) was washed twice in ultrapure water and autoclaved to sterilise. Equal volumes of 2× SLAD formulation and 4% molten agar were combined and 20 ml aliquots poured into Petri dishes.

Four colonies of *S*. *cerevisiae* strain AWRI796 were seeded on each plate at different distances from the centre, each initially containing approximately 1000 cells. A well was cut into the centre of each plate using a pipette tip, into which 5 μl of molten agar was added and left to solidify. Following this, 5 μl of the nutrient source was added, resulting in the same amount of nutrient added to the plate as in the unrestricted case. The colonies were grown for 16 days at 30 °C and digital images were taken with a GXCAM HiChrome-S camera mounted on to a Leica S8 APO stereo microscope.

### Code availability

The computer codes used in the current study are available from the corresponding author upon reasonable request.

### Data availability

The data that support the findings of this study are available from the corresponding author upon reasonable request.
